# Effect of “maccog” TCM tea on improving glucolipid metabolism and gut microbiota in patients with type 2 diabetes in community

**DOI:** 10.3389/fendo.2023.1134877

**Published:** 2023-03-08

**Authors:** Biyue Hu, Tongtong Yin, Jiajia Zhang, Minjing Liu, Hang Yun, Jian Wang, Renmei Guo, Jie Huang, Yixia Zhou, Hongyan Meng, Li Wang

**Affiliations:** ^1^ Cardiovascular Department, The Frist Affiliated Hospital of Soochow University, Suzhou, China; ^2^ School of Nursing, Suzhou Medical College of Soochow University, Suzhou, China; ^3^ Research Center, Soochow Setek Biotechnology Co, Ltd, Suzhou, China; ^4^ Nursing School of Guizhou University of Traditional Chinese Medicine (TCM), Guizhou, China

**Keywords:** type 2 diabetes mellitus, traditional chinese medicine, medicine food homology, blood glucose, gut microbiota

## Abstract

**Objectives:**

This work aimed to observe the effect of consuming Chinese herb tea on glucolipid metabolism and gut microbiota in patients with type 2 diabetes mellitus (T2DM).

**Methods:**

Ninety patients with T2DM were recruited from a community and randomly divided into the control group (CG) and intervention group (IG). CG maintained conventional treatment and lifestyle, and IG accepted additional “maccog” traditional Chinese medicine (TCM) tea (mulberry leaf, radix astragali, corn stigma, cortex lycii, radix ophiopogonis, and gynostemma) for 12 weeks. Glucolipid metabolism, hepatorenal function, and gut microbiota were then measured.

**Results:**

After the intervention, the decreases in fasting plasma glucose (FPG) and total cholesterol (TC) were greater (P<0.05) in IG than in CG, and those in glycosylated serum protein (GSP) were almost significantly greater (P=0.066) in IG than in CG. The total protein (TP), albumin (ALB), and creatinine (CREA) levels in IG were significantly lower and their decreases were larger in IG than in CG (P<0.05) after the intervention. The Ace and Chao1 indices in IG were slightly higher after the intervention (P=0.056 and 0.052, respectively) than at baselines. The abundance of *Actinobacteria*, *Lachnospiraceae*, *Bifidobacteriaceae*, and *Phascolarctobacterium* increased significantly after the intervention in IG (P<0.05), and the abundance was higher in IG than in CG (P<0.05 or P<0.1). The abundance of *Clostridiales* and *Lactobacillales* was negatively correlated with FPG (P<0.05), *Clostridiales* and *Lachnospiraceae* was negatively correlated with GSP (P<0.05), and *Bacteroides*/*Firmicutes* was positively correlated with both (P<0.05). No adverse event was observed during the intervention.

**Conclusions:**

Administration of “maccog” TCM tea for 12 weeks slightly improved glucolipid metabolism and significantly increased the abundance of beneficial gut microbiota in community patients with T2DM. The increase in beneficial bacteria abundance may be involved in the improvement of glucose metabolism indicators. In addition, this intervention is safe and feasible.

**Clinical trial registration:**

https://www.chictr.org.cn/showproj.aspx?proj=31281, identifier ChiCTR1800018566.

## 1 Introduction

Type 2 diabetes mellitus (T2DM) is a metabolic disorder disease characterized by chronic hyperglycemia. Its main pathogenesis includes insulin resistance and insulin secretion disorders leading to absolute insulin deficiency (1). The continued growth of patients with T2DM has led to a serious public health problem (2). Its clinical treatment mainly depends on the long-term use of medicine to control the disease. Although treatment can have certain positive effects, a decrease in drug effect, an increase in adverse reaction, and potential complications may occur over time (3).

Compared with conventional hypoglycemic drugs, traditional Chinese medicine (TCM) may have lower cost and fewer side effects and delay the occurrence and development of diabetes complications through the overall regulation of the human body (4). Many animal studies have shown that mulberry leaves, gynostemma, radix astragalus, and radix ophiopogonis can improve insulin sensitivity, stimulate insulin secretion, protect pancreatic islet function, inhibit intestinal carbohydrate intake, promote lipid metabolism, and inhibit peroxidation to achieve hypoglycemic, hypolipidemic, and anti-oxidative effects (5–8). These effects may play an important role as effective supplements and alternatives to conventional antidiabetic drugs. However, such studies in human subjects are lacking. In addition, medicine food homology (MFH) is provided as decoction in most studies (9, 10). Although it may have certain effects on controlling blood glucose, the method is relatively complicated and inconvenient to carry out, which is not convenient for long-term auxiliary hypoglycemic use.

Comparatively speaking, taking Chinese herbs as tea fits with the daily tea drinking habits of the Chinese. It enables the integration of nutritional health care into daily drinking, which causes minimal mental burden and time cost. As a result of fewer ingredients extracted from tea and consumed by human beings compared with decoction, TCM tea should be much safer (but also may be less effective, and this point may be compensated by adopted a longer period). Given their simplicity, convenience, acceptability, and wide applicability, TCM teas have received increasing attention in health care for chronic diseases.

In recent years, studies have pointed out that the gut microbiota are closely related to the occurrence and development of T2DM. Intestinal flora participates in human metabolism, affects the energy balance, and regulates blood sugar levels and chronic inflammatory reaction (11). If dysbacteriosis appears, it will produce immunotoxins, reduce the intestinal barrier function, increase intestinal permeability, promote metabolic endotoxemia, and trigger chronic inflammation, leading to obesity, insulin resistance, diabetes, and other diseases (12).

According to research findings, there are significant differences in gut microbiota between diabetics and nondiabetics. Compared with normal people, the number of *Bifidobacterium*, *Prevotella*, *Clostridium coccoides*, and *Atopobium cluster* in the gut microbiota of diabetics decreases significantly, whereas the number of *Lactobacillus* increases significantly (13, 14). Moderate alterations in intestinal flora have been observed in patients with T2DM (15). In addition, the abundance of *Bacteroides* in the T2DM group is only half that of the normal glucose tolerance (NGT) and prediabetes (pre-DM) groups, and *Verrucomicrobiae* has a significantly lower abundance in both the pre-DM and T2DM groups (16). Furthermore, a correlation was found between different gut microbes and metabolic parameters. Others have shown that the amount of *Faecalibacterium* in the intestine of patients with T2DM is negatively correlated with levels of FPG, HbA1c, and 2h-postprandial blood glucose but is positively correlated with homeostatic model assessment of β-cell function (17).

In summary, changes in the structure of the intestinal flora are likely to be an important factor affecting T2DM, or even a target to control the development of the disease. Previous studies suggested that food formulas based on whole grains, TCM foods, and prebiotics can help patients with obesity improve blood pressure, lipid profile, and insulin sensitivity. Meanwhile, they can significantly reduce opportunistic pathogens, such as *Enterobacteriaceae* and *Desulfovibrionaceae*, and increase gut barrier-protecting *Bifidobacterium* spp (9). The combination of metformin and traditional Chinese herbal formulas can significantly ameliorate hyperglycemia and hyperlipidemia in T2DM patients with hyperlipidemia, as well as change the structure of intestinal flora (18). In addition, Gegen Qinlian Decoction (GQD) can significantly reduce FPG and HbA1c levels and enrich the amounts of *Faecalibacterium*, *Bifidobacterium*, and *Gemmiger* (10). All of these results suggest that the changes in structure of gut microbiota may be a potentially effective adjunctive treatment for diabetes.

Therefore, the purpose of this study was to explore the effect of “maccog” TCM tea (mulberry leaf, radix astragali, corn stigma, cortex lycii, radix ophiopogonis, and gynostemma) on improving blood glucose and gut microbiota of patients with T2DM. A 12-week randomized controlled intervention trial was conducted, and the internal relationship between blood glucose and gut microbiota was analyzed. The results are expected to provide reference for the adjuvant treatment of patients with T2DM and research about gut microbiota.

## 2 Methods

### 2.1 Sample size estimate

The sample size was estimated with an estimation formula for a two-group comparison with a random design:


$${\text{n}}_{1}={\text{n}}_{2}=2\left[\frac{(z_{\alpha }+z_{\beta })\sigma }{\delta }\right]2$$

Z_α_: the corresponding Z value for type I error α; Z_β_: the corresponding Z value for type II error β; σ: standard deviation; and δ: permissible error

The unilateral test was adopted with α=0.05, β=0.10, Z_α_=1.645, and Z_β_=1.282. HbA1c was considered the primary outcome indicator. We concluded X_1 =_ 7.4%, X_2 =_ 8.1%, δ=0.7, and σ=1.0 by reviewing the related literature (19). The calculated result was n_1_=n_2 =_ 36. Assuming an attrition rate of 20%, each group needed 44 patients, and a minimum of 88 patients was required.

### 2.2 Participants

All the participants were recruited from the Canglangting Healthcare Community of Suzhou. Patients with T2DM were included in the study (aged 40–80 years; receiving diet control or medication for more than 4 weeks). The exclusion criteria were as follows: had severely diseased heart, liver, kidney, brain, tumor, and acute diabetic complications; receiving other forms of dietary therapy; and allergic to medicine and food homologous substances or other allergic constitutions. This study was approved by the Ethics Committee of Soochow University (ECSU-201800076). This study conformed to the provisions of the Declaration of Helsinki and was registered with the Chinese Clinical Trial Registry (ChiCTR1800018566). Informed consent was obtained from all participants.

### 2.3 Study procedure

The study was a 12-week randomized controlled clinical trial. We randomly divided participants into two groups. First, a random number for each participant was made through SPSS software (IBM, Armonk, NY, USA), and the numbers of participants were sorted and included in the control group (CG, N=45) and the intervention group (IG, N=45). CG maintained the conventional treatment and the original lifestyle, and IG was administered with “maccog” TCM tea. The formula of “maccog” TCM tea included 2 g of mulberry leaf, 2 g of radix astragali, 2 g of corn stigma, 2 g of cortex lycii, 2 g of gynostemma, and 3 g of radix ophiopogonis, totaling 13 g. A procedure for the formation of the “maccog” TCM tea formula was added in the supplementary material. The “maccog” TCM tea was soaked in hot water for about 10 min, and participants drank the water when it became warm. The water could be refilled, and the recommended total volume was 1000–1500 mL every day. The prescribed total days were 6–7 days a week successively for 12 weeks. All the herbs were purchased from Suzhou Tianling Traditional Chinese Medicine Pieces Co., Ltd. (brand Li Liangji). During the intervention period, we required all participants to maintain their usual diet and lifestyles and provided no other health guidance.

### 2.4 Measurements

The primary outcomes included changes in fasting plasma glucose (FPG), glycosylated serum protein (GSP), glycated hemoglobin (HbA1c), blood lipids, and gut microbiota. The secondary outcomes included changes in hepatorenal function indicators, abnormal complaints, and adverse reactions. The FPG and GSP were measured before the intervention (baseline) and at 6 and 12 weeks after the intervention. The HbA1c, blood lipids, hepatorenal function, and gut microbiota were measured at baseline and week 12. The changes in medication, any adverse reactions, and abnormal complaints were recorded during the intervention.

FPG was determined by using a Contour TS blood glucose meter and supporting test strips (BAYER, Germany). GSP was measured with a microplate reader (Multiskan™ FC, Finland). HbA1c was tested with an automatic special protein dry immunochromatographic analyzer (AS100, Alere Technologies AS, Oslo, Norway). Blood lipids, including total cholesterol (TC), triacylglycerol (TG), high-density lipoprotein (HDL), and low-density lipoprotein (LDL), and hepatorenal function indicators, including alanine aminotransferase (ALT), aspartate aminotransferase (AST), total protein (TP), albumin (ALB), total bilirubin (TBIL), alkaline phosphatase (ALP), blood urea nitrogen (BUN), creatinine (CREA), and uric acid (UA), were detected with an automatic chemistry analyzer (7100, Hitachi, Tokyo, Japan).

Stool samples were kept frozen at −80°C until testing. DNA was extracted using the QIA amp Power Fecal DNA Kit (REF: 12830-50, QIAGEN, Germany). Fecal DNA was amplified by PCR using 16S amplicon PCR forward primer and 16S amplicon PCR reverse primer. The amplicons were pooled and sequenced by the HiSeq 2000 platform. Sequences were clustered into operational taxonomic units (OTUs) based on the SILVA128 database, at a similarity level of 97%. Alpha and beta diversities were calculated using Quantitative Insights Into Microbial Ecology (QIIME) and R (version 3.2.0).

Meanwhile, the participants completed a self-designed questionnaire on basic information. Among the questions investigated, regular exercise referred to the performance of exercise of no less than three times a week, with each instance lasting for no less than 30 min (20). Physical activity referred to occupational, household, transportation, and other daily activities (21). Patients chose the appropriate options according to their actual situations.

### 2.5 Statistical analysis

Statistical analyses were carried out by IBM SPSS Statistic 26.0. Origin 2018 was used to draw diagrams. The Shapiro–Wilk test was used to verify the normal distribution of data. The data are expressed as the mean ± SD or M (P25, P75) unless specifically noted. Two-way repeated measures ANOVA was used to compare the data in CG and IG from different time points. The independent sample t-test, Mann–Whitney U test, and chi-square test were used to compare the changes (Δ, equals to week 12–week 0) after the intervention between the two groups. Correlations were assessed by Pearson or Spearman rank correlation analysis. P<0.05 was set as statistically significant. Alpha and beta diversities were generated in QIIME and calculated based on weighted unifrac distance matrices.

## 3 Results

### 3.1 Basic characteristics of participants

**Figure 1:**
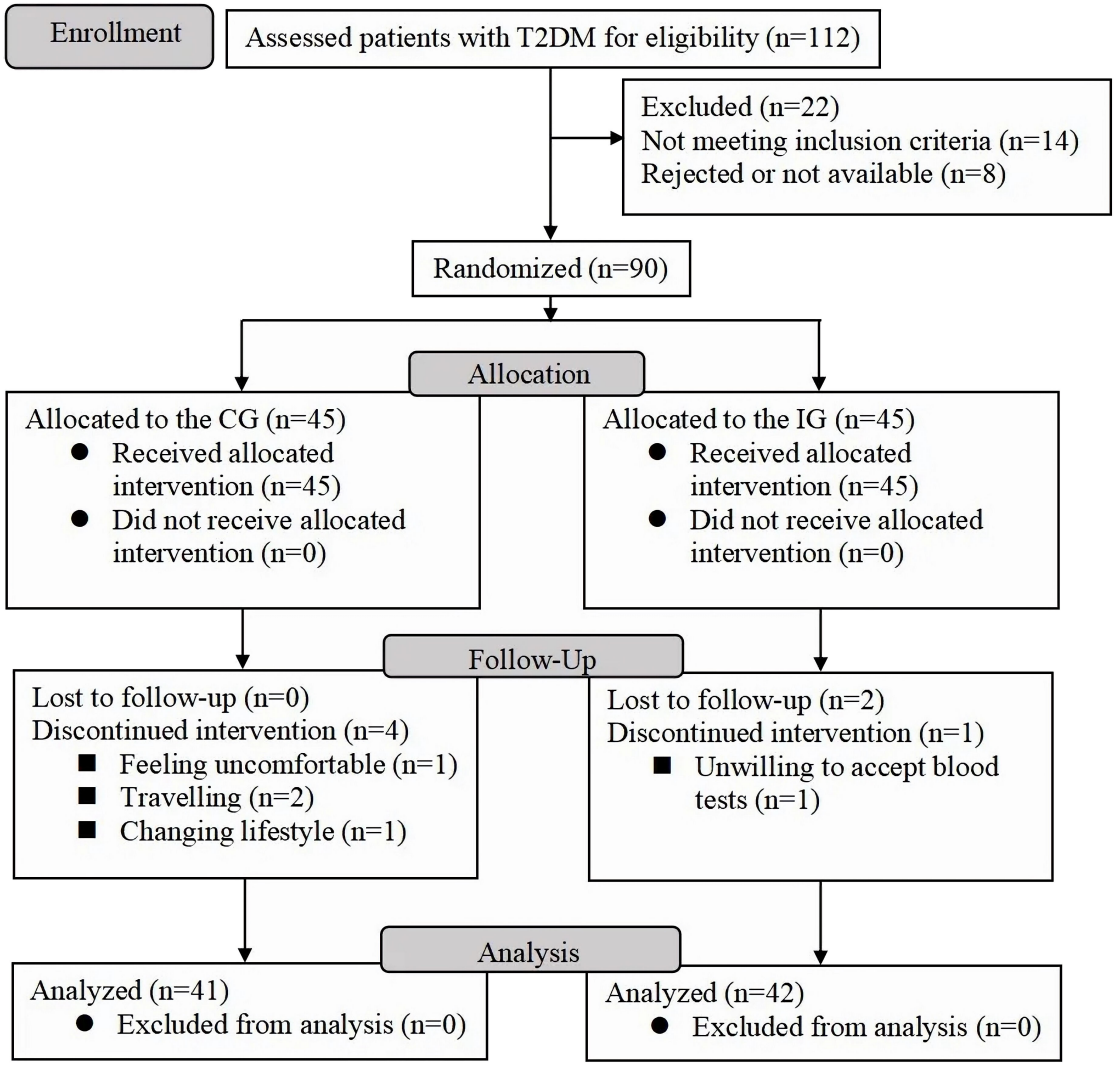
Participant flowchart. CG, control group; IG, intervention group.

A total of 112 patients with T2DM were screened, and 90 of them who met the inclusion criteria were randomly divided into IG and CG (45 cases in each). Finally, 83 participants (41 in IG, aged 70.7 ± 5.5 years and 42 in CG, aged 71.6 ± 4.7 years) were included in the statistical analysis (**Figure 1**). No significant differences in baseline characteristics and clinical parameters (**Table 1**) were found between IG and CG.

**Table 1:** Basic Characteristics of Participants, (mean ± SD) or n (%).

	Total	CG	IG	t/Z/χ^2^	P
**Number**	83 (100)	42 (50.6)	41 (49.4)		
**Age (years)**	71.1 ± 5.1	71.6 ± 4.7	70.7 ± 5.5	-0.744	0.459^a^
**Height (cm)**	163.1 ± 8.5	163.2 ± 8.7	163.0 ± 8.4	-0.090	0.928^a^
**Weight (kg)**	65.7 ± 11.7	66.9 ± 11.7	64.4 ± 11.6	-1.010	0.315^a^
**BMI (kg/m^2^)**	24.5 ± 3.2	25.0 ± 3.6	24.0 ± 2.8	-1.404	0.164^a^
**SBP (mmHg)**	141.1 ± 13.4	142.6 ± 13.9	139.5 ± 12.9	-1.065	0.290^a^
**DBP (mmHg)**	78.1 ± 9.6	78.6 ± 9.6	78.0 ± 9.2	-0.325	0.746^a^
**Heart rate (beats/min)**	78.7 ± 8.2	79.5 ± 9.1	78.0 ± 7.3	-0.827	0.410^a^
**Gender**				0.120	0.729^c^
Males, n (%)	47 (56.6)	23 (48.9)	24 (51.1)		
Females, n (%)	36 (43.4)	19 (52.8)	17 (47.2)		
**Diabetes course (years)**	10.0 (4.0, 17.0)	9.5 (4.0, 15.0)	13.0 (4.0, 18.5)	-0.775	0.438^b^
**Diabetes complications, n (%)**				1.359	0.954^d^
Retinopathy	21 (55.3)	11 (55.0)	10 (55.6)		
Nephropathy	2 (5.3)	1 (5.0)	1 (5.6)		
Neuropathy	6 (15.8)	3 (15.0)	3 (16.7)		
Cardiovascular disease	6 (15.8)	4 (20.0)	2 (11.1)		
Cerebrovascular disease	3 (7.9)	1 (5.0)	2 (11.1)		
**Clinical treatment, n (%)**				1.923	0.618^d^
No	10 (12.0)	4 (9.5)	6 (14.6)		
Drug	56 (67.5)	31 (73.8)	25 (61.0)		
Insulin	6 (7.2)	3 (7.1)	3 (7.3)		
Drug plus insulin	11 (13.3)	4 (9.5)	7 (17.1)		
**Smoking, n (%)**				0.122	0.727^c^
No	70 (84.3)	36 (85.7)	34 (82.9)		
Yes	13 (15.7)	6 (14.3)	7 (17.1)		
**Drinking, n (%)**				0.186	0.666^c^
No	61 (73.5)	30 (71.4)	31 (75.6)		
Yes	22 (26.5)	12 (28.6)	10 (24.4)		
**Regular exercise, n (%)**				1.014	0.314^c^
No	28 (33.7)	12 (28.6)	16 (39.0)		
Yes	55 (66.3)	30 (71.4)	25 (61.0)		
**Physical activity, n (%)**				0.002	0.964^c^
Light	12 (14.5)	6 (14.3)	6 (14.6)		
Moderate	71 (85.5)	36 (85.7)	35 (85.4)		

a , independent sample t-test;

b , Mann–Whitney U test;

c , chi-square test;

d , Fisher’s exact test; SD, standard deviation; CG, control group; IG, intervention group; BMI, body mass index; SBP, systolic blood pressure; DBP, diastolic blood pressure.

### 3.2 Compliance

The results showed that 73.2% of the participants had an average daily consumption dose of 1000–1500 mL, and 85.4% of the participants consumed the “maccog” TCM tea for 6–7 days per week, which met the recommended volume and frequency (**Table 2**).

**Table 2:** Actual consumption dose and days in the intervention group.

		Number of participants	Percentage (%)
**Average daily consumption dose (mL)**	>1500	7	17.1
	1000–1500	30	73.2
	500–1000	4	9.7
	Total	41	100
**Average weekly consumption day (days/week)**	6–7	35	85.4
	4–5	6	14.6
	1–3	0	0
	Total	41	100

### 3.3 Efficacy of “maccog” TCM tea for glycemic control

At baseline, there was no group difference in FPG and HbA1c, but GSP was higher in IG than in CG. Post-intervention levels of FPG and GSP showed no group difference (P>0.05). The decrease in FPG at week 12 in IG was larger than that in CG (P=0.033), and the decrease in GSP at week 12 in IG was almost significantly greater than that in CG (P=0.066). GSP in IG was significantly lower after 6 weeks and 12 weeks of intervention compared with that pre-intervention (P<0.01). Neither the pre- and post-intervention levels nor the changes in HbA1c showed group differences (P>0.05) (**Table 3**).

**Table 3:** Changes in blood glucose indicators in the two groups, (mean ± SD) or *M (P_25_, P_75_)*.

Indicators	Group	Time points			*Z/P* ^b^	*F_t_/P_t_*	*F_g_/P_g_*	*F_i_/P_i_*	△
		Pre	Week 6	Week 12					
**FPG**	CG	7.55 ± 2.09	7.74 ± 1.85	7.70 ± 1.75		0.471/0.598	1.004/0.322	2.338/0.103	0.30 (-0.40,1.03)
	IG	8.32 ± 2.67	7.99 ± 2.39	7.60 ± 1.95					-0.40 (-1.65, -0.80)
	*Z*								-2.133
	*P^a^*								0.033
**GSP**	CG	3.22 ± 0.35	2.94 ± 0.29†	2.81 ± 0.28††		86.420/<0.01	2.191/0.147	4.672/0.016	-0.35 (-0.67, -0.18)
	IG	3.43 ± 0.56	3.03 ± 0.38†	2.83 ± 0.37††					-0.47 (-0.92, -0.25)
	*F*	4.220	1.662	0.056					-1.840
	*P*	0.047	0.210	0.815					0.066
**HbA1c**	CG	7.0 (6.4, 7.7)	/	6.9 (6.2, 7.8)	-1.031/0.303				-0.2 (-0.2, 0.3)
	IG	7.0 (6.4, 8.6)	/	6.9 (6.5, 8.7)	-0.598/0.550				0.0 (-0.4, 0.3)
	*Z*	-0.515		-0.647					-0.394
	*P* ^a^	0.606		0.517					0.694

a Mann–Whitney U test;

b Wilcoxon signed rank test; *F_t_/P_t_, F_g_/P_g_*, and *F_i_/P_i_*: two-way repeated measures ANOVA, time effect, group effect and group. *time interaction;

†: P<0.05 vs. Pre; ††: P<0.05 vs. Week 6; CG, control group; IG, intervention group; SD, standard deviation; △, value at week 12 minus value at pre.

### 3.4 Efficacy of “maccog” TCM tea for blood lipid profile

No significant differences were found in blood lipid levels between the two groups at baseline. TC and LDL in IG were significantly lower after 12 weeks of the intervention compared with those before the intervention (P<0.05). TC was significantly lower in IG than in CG at week 12 (P<0.05). The decrease of TC in IG was larger than that in CG. HDL/LDL in both groups significantly increased after the intervention (P<0.05) (**Table 4**).

**Table 4:** Changes in blood lipid levels in two groups (mean ± SD).

		CG	IG	*F_t_/P_t_*	*F_g_/P_g_*	*F_i_/P_i_*
**TC (mmol/L)**	Pre	4.60 ± 0.93	4.49 ± 1.04	29.181/<0.01	5.060/0.030	20.053/<0.01
	Week 12	4.49 ± 0.93	3.84 ± 0.92†^*^			
	*F*	1.510	48.484			
	*P*	0.226	<0.01			
	△	-0.11 ± 0.58	-0.65 ± 0.60†	4.167/<0.01		
**TG (mmol/L)**	Pre	1.78 ± 1.33	1.52 ± 1.18	2.569/0.117	1.429/0.239	0.413/0.524
	Week 12	1.71 ± 1.11	1.34 ± 0.76			
	△	-0.07 ± 0.91	-0.18 ± 0.80	0.573/0.568		
**HDL (mmol/L)**	Pre	1.25 ± 0.32	1.32 ± 0.35	0.295/0.590	0.063/0.802	3.634/0.064
	Week 12	1.32 ± 0.37	1.29 ± 0.38			
	△	0.07 ± 0.17	-0.04 ± 0.35	1.752/0.085		
**LDL (mmol/L)**	Pre	2.69 ± 0.85	2.75 ± 0.68	11.403/<0.001	0.085/0.772	4.351/0.043
	Week 12	2.55 ± 0.81	2.38 ± 0.78^*^			
	*F*	3.995	11.655			
	*P*	0.052	<0.01			
	△	-0.14 ± 0.47	-0.36 ± 0.68	1.702/0.093		
**HDL/LDL**	Pre	0.52 ± 0.23	0.51 ± 0.20	13.706/<0.001	0.115/0.736	1.154/0.289
	Week 12	0.57 ± 0.25^*^	0.62 ± 0.32^*^			
	*F*	18.561	6.515			
	*P*	<0.01	0.015			
	△	0.06 ± 0.09	0.11 ± 0.26	-1.059/0.295		

*F_t_/P_t_, F_g_/P_g_*, and *F_i_/P_i_*: two-way repeated measures ANOVA, time effect, group effect, and group, *time interaction;

†: P<0.05 vs. CG; ^*^: P<0.05 vs. Pre; CG, control group; IG, intervention group.

### 3.5 Efficacy of “maccog” TCM tea for hepatorenal function parameters

There was no significant difference between the two groups at baseline. The TP, ALB, and CREA levels at week 12 were significantly lower in IG than in CG (P<0.01). The decrease in AST, TP, ALB, and CREA was larger in IG than in CG at week 12 (P<0.05). The AST, TP, ALB, ALP, and CREA levels in IG were significantly lower after 12 weeks of the intervention compared with those before the intervention (P<0.05). The levels of all indicators were within normal range (**Table 5**).

**Table 5:** Changes in hepatorenal levels in two groups (mean ± SD).

		CG	IG	*F_t_/P_t_*	*F_g_/P_g_*	*F_i_/P*_i_
**ALT (U/L)**	Pre	21.07 ± 10.17	20.50 ± 5.70	0.601/0.443	0.666/0.419	0.827/0.369
	Week 12	21.26 ± 10.62	19.03 ± 8.28			
	△	0.19 ± 7.59	-1.47 ± 8.18	0.958/0.341		
**AST (U/L)**	Pre	18.50 ± 7.03	20.27 ± 6.90	10.674/<0.01	0.131/0.719	3.716/0.061
	Week 12	18.10 ± 6.77	17.47 ± 4.25^*^			
	*F*	0.341	10.421			
	*P*	0.563	<0.01			
	△	-0.40 ± 4.49	-2.80 ± 5.55†	2.161/0.034		
**TP (g/L)**	Pre	71.33 ± 5.06	69.66 ± 4.44	1.331/0.256	10.502/<0.01	6.180/0.017
	Week 12	72.24 ± 4.29	67.35 ± 5.42†^*^			
	*F*	1.110	6.718			
	*P*	0.298	0.013			
	△	0.91 ± 5.60	-2.31 ± 5.70†	2.595/0.011		
**ALB (g/L)**	Pre	44.80 ± 3.26	43.72 ± 3.17	5.138/0.029	14.507/<0.01	19.719/<0.01
	Week 12	45.31 ± 3.61	41.91 ± 3.47†^*^			
	*F*	2.604	16.283			
	*P*	0.114	<0.01			
	△	0.50 ± 2.02	-1.81 ± 2.88†	4.236/<0.01		
**TBIL (µmol/L)**	Pre	9.69 ± 4.09	10.29 ± 2.28	2.754/0.105	0.033/0.857	1.608/0.212
	Week 12	10.82 ± 5.03	10.48 ± 2.75			
	△	1.12 ± 4.50	0.19 ± 2.34	1.186/0.240		
**ALP (U/L)**	Pre	100.12 ± 21.14	101.10 ± 18.53	26.841/<0.01	0.705/0.406	1.765/0.192
	Week 12	94.76 ± 21.80	88.02 ± 17.41^*^			
	*F*	2.552	14.696			
	*P*	0.118	<0.01			
	△	-5.36 ± 21.73	-13.07 ± 21.84	1.613/0.111		
**BUN (mmol/L)**	Pre	5.75 ± 1.23	5.53 ± 0.82	0.038/0.846	<0.01/0.995	2.053/0.160
	Week 12	5.61 ± 1.42	5.76 ± 1.01			
	△	-0.15 ± 1.23	0.22 ± 1.24	-1.357/0.179		
**CREA (µmol/L)**	Pre	74.06 ± 15.77	75.58 ± 15.23	24.203/<0.01	2.408/0.129	19.456/<0.01
	Week 12	73.98 ± 16.06	63.41 ± 13.60†^*^			
	*F*	0.006	26.043			
	*P*	0.940	<0.01			
	△	-0.08 ± 6.92	-12.17 ± 15.26†	4.626/<0.01		
**UA (µmol/L)**	Pre	328.08 ± 70.56	323.93 ± 84.13	0.012/0.913	0.041/0.840	2.777/0.103
	Week 12	320.91 ± 69.92	330.83 ± 66.91			
	△	-7.16 ± 39.83	6.89 ± 40.35	-1.597/0.114		

*F_t_/P_t_*, *F_g_/P_g_*, and *F_i_/P_i_*: two-way repeated measures ANOVA, time effect, group effect, and group. *time interaction;

†: P<0.05 vs. CG; *: P<0.05 vs. Pre; CG, control group; IG, intervention group.

### 3.6 Efficacy of “maccog” TCM tea for gut microbiota

The overall microbiota structure at the phylum level in IG and CG before and after the intervention is presented in **Figure S1-A** in supplementary material. The main phyla of IG and CG were *Bacteroidetes*, *Firmicutes*, *Proteobacteria*, and *Actinobacteria*, with *Bacteroidetes* being the most abundant (**Figure S1-A**). To compare the overall gut microbiota structure in participants, principal coordinate analysis (PCoA) according to OTUs of each sample was implemented to provide a glimpse of gut microbial dynamics between CG and IG. The weighted results of PCoA were PC1 = 38.66% and PC2 = 18.51% of total variations (**Figure S1-B**).

**Figure 2:**
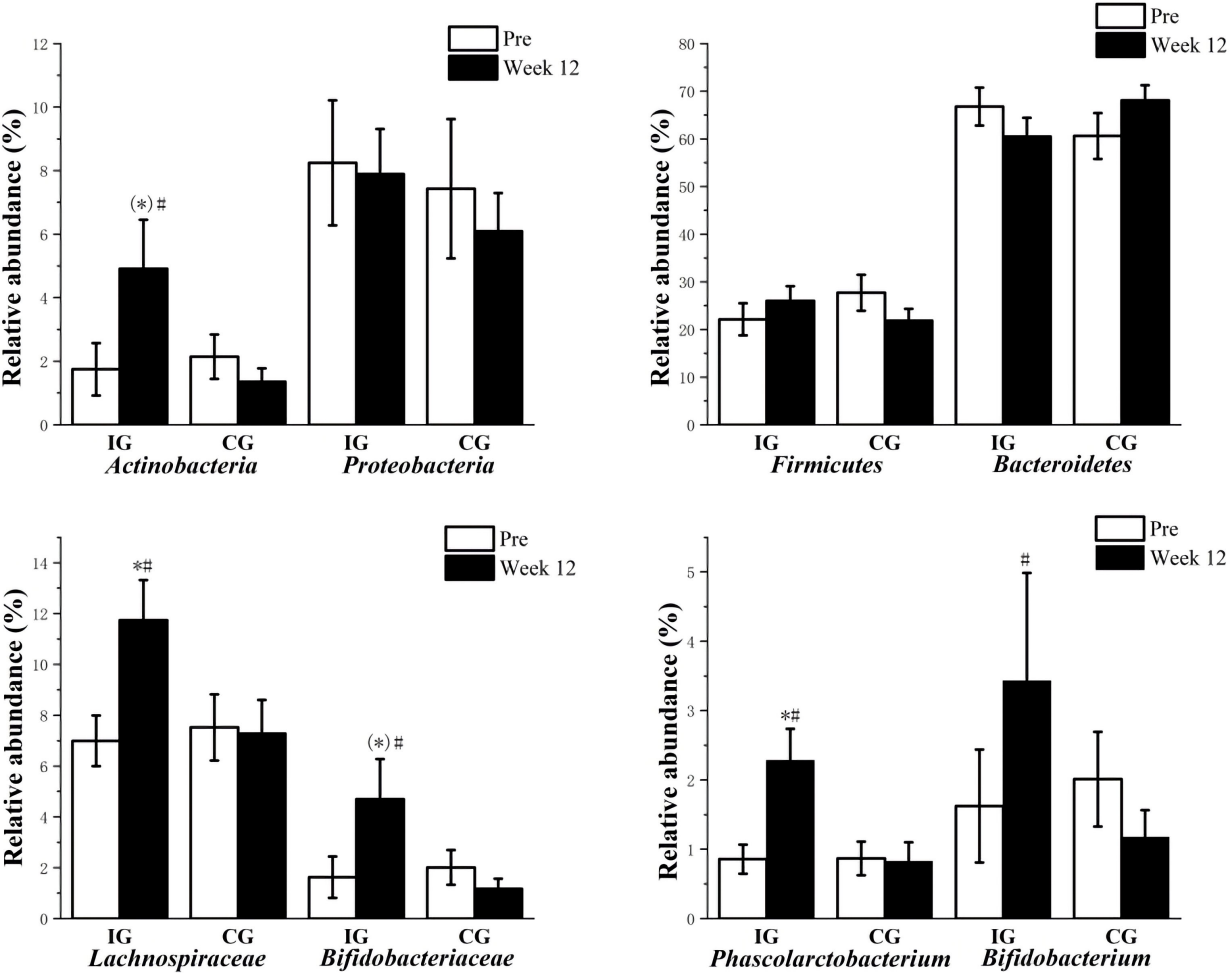
Changes in gut microbiota in two groups (mean ± SEM). ^#^: *P*<0.05 vs. Pre, ^*^: *P*<0.05 vs. CG, (^*^): 0.05<*P*<0.1 vs. CG; SEM, standard error of mean; CG, control group; IG, intervention group.

No significant difference was noted between the two groups at baseline. The Ace and Chao1 indices in IG were slightly higher after the intervention, and the values were close to significant differences (P=0.056, P=0.052; **Table 6**). The abundance of *Actinobacteria*, *Lachnospiraceae*, *Bifidobacteriaceae*, *Phascolarctobacterium*, and *Bifidobacterium* after the intervention was significantly higher in IG than that at baseline (P<0.05). The abundance of *Lachnospiraceae* and *Phascolarctobacterium* after the intervention was significantly higher in IG than in CG (P<0.05) (**Figure 2**).

**Table 6:** Changes in the Alpha diversity indices in two groups (mean ± SD).

	time	CG	IG	*F_t_/P_t_*	*F_g_/P_g_*	*F_i_/P*_i_
**Ace**	Pre	218.13 ± 91.41	201.81 ± 92.07	2.121/0.157	0.001/0.982	1.275/0.269
	Week 12	230.94 ± 96.90	239.00 ± 105.21			
	*F*	0.253	3.958			
	*P*	0.619	0.056			
	△	12.81 ± 134.71	37.19 ± 102.39	-0.779/0.439		
**Chao1**	Pre	211.05 ± 89.73	195.98 ± 90.35	2.293/0.142	0.015/0.903	1.324/0.260
	Week 12	224.96 ± 97.26	234.47 ± 105.53			
	*F*	0.308	4.119			
	*P*	0.583	0.052			
	△	13.91 ± 132.55	38.48 ± 103.85	-0.789/0.434		
**Shannon**	Pre	4.04 ± 1.05	3.95 ± 1.24	0.061/0.807	0.269/0.608	2.069/0.162
	Week 12	3.90 ± 1.04	4.15 ± 0.96			
	△	-0.15 ± 1.36	0.20 ± 1.11	-1.066/0.291		
**Simpson**	Pre	0.82 ± 0.13	0.81 ± 0.19	0.052/0.821	0.195/0.662	1.703/0.203
	Week 12	0.80 ± 0.17	0.84 ± 0.10			
	△	-0.02 ± 0.20	0.03 ± 0.17	-1.034/0.306		

*F_t_/P_t_, F_g_/P_g_*, and *F_i_/P_i_*: two-way repeated measures ANOVA, time effect, group effect, and group. *time interaction; CG, control group; IG, intervention group.

### 3.7 Correlation between gut microbiota abundance and glucose metabolism indicators

The abundance of *Clostridiales* and *Lactobacillales* was negatively correlated with FPG levels (P<0.05), and the abundance of *Clostridiales* and *Lachnospiraceae* was negatively correlated with GSP levels (P<0.05). The abundance of *Bacteroides*/*Firmicutes* was positively correlated with FPG and GSP levels (P<0.05) (**Table 7**).

**Table 7:** Correlation between gut microbiota abundance and glucose metabolism indicators.

	FPG		GSP		HbA1c	
	r	P	r	P	r	P
***Clostridiales***	-.197^*^	.034	-.221^*^	.017	-.154	.099
***Lachnospiraceae***	-.117	.212	-.188^*^	.043	-.076	.419
***Lactobacillales***	-.204^*^	.028	-.178	.056	-.136	.145
***Bacteroides/Firmicutes***	.200^*^	.031	.237^*^	.010	.156	.094

*P<0.05.

### 3.8 Changes in diet and medication during the intervention

One participant in CG had changes in the diet during the study, which was more frequent eating outside and the resulting increased intake of greasy food. In IG, there were no significant changes in diet or other lifestyle behaviors. The medication (for diabetes) doses of two patients decreased and that of one increased in IG, and those of two patients decreased in CG. Medication doses were adjusted either by the patients (on the basis of self-monitored blood glucose levels) or their doctors (**Table 8**).

**Table 8:** Changes in medication during the intervention.

Group	Change	n	Details
**IG**	Increase	1	(1) Laideshi insulin 14U/day→15U/day (doctor’s prescription)
	Decrease	2	(1) Youmile insulin 19U/day→17U/day (self-adjustment)
			(2) Yamoli 1 mg/day→0.5 mg/day (doctor’s prescription)
**CG**	Increase	0	
	Decrease	2	(1) Novoline 25U →20/25U in the morning, and 15U→10U in the evening (self-adjustment)
			(2) Metformin 0.25 g/day → withdrawal (doctor’s prescription)

CG, control group; IG, intervention group.

### 3.9 Adverse reactions and physical changes

During the intervention, no hypoglycemia was reported. In IG, one participant reported skin itching, three reported xerostomia, five reported improvement in vigor, six reported defecation increased, five reported stool form improvement, five reported sleep quality improvement, and two reported remarkable improvement with blurred vision. In CG, two participants reported vigor improvement and three reported an increase in defecation.

## 4 Discussion

By providing “maccog” TCM tea to patients with T2DM, our research found some minor positive effects of such an intervention on glucolipid metabolism and gut microbiota and proved its safety and feasibility.

The minor positive effects in glycemic control were demonstrated by the significantly larger decrease in FPG and the slightly larger reduction of GSP in IG compared with CG. This finding was similar to the results of Huyen and Chatterji’s clinical studies (19, 22). After 12 weeks of gynostemma pentaphyllum tea intervention in patients with T2DM, Huyen et al. found that the FPG levels in the experimental group significantly decreased compared with those in the placebo group (19). After 12 weeks of compound capsule (containing *Morus alba, Artemisia dracunculus, Urtica dioica, Cinnamomum zeylanicum, and Taraxacum officinale*) intervention in patients with T2DM, Chatterji et al. found that the FPG levels of participants significantly decreased compared with those at baseline (22). In the mechanism by which TCM affects glucose metabolism, previous studies have shown that mulberry leaf polysaccharide effectively normalizes hepatic glucose metabolism and insulin signaling by inhibiting the expression of protein–tyrosine phosphatase 1B and mitigating oxidative stress in the livers of rats with type 2 diabetes (23). A polysaccharide extracted from gynostemma pentaphyllum revealed excellent capacity in inhibiting α-glucosidase activity and glucose absorption and curing diabetic mice (5). *Astragalus membranaceus* improves glucose metabolism and insulin sensitivity in T2DM by directly enhancing insulin-stimulated glucose uptake in insulin-resistant myotubes with improved insulin signalling and inflammatory response and oxidative stress (24). Polysaccharides from corn silk exert appreciable hypoglycemic activity by inhibiting α-amylase and α-glucosidase and enhancing glucose uptake in rat L6 skeletal muscle cells (25). *Ophiopogon japonicus* extract can significantly lower blood glucose levels on experimental type 2 diabetic rats by improving insulin sensitivity and increasing glycogen contents in liver and skeletal muscle (26). Taurine in the cortex lycii can reduce the lipid peroxidation level under hyperglycemia and protect β-cells, and the polysaccharides from cortex lycii can inhibit α-glucosidase and lower the glucose absorption rate (27). In this study, the combined use of these substances for 12 weeks significantly decreased FPG levels and slightly decreased GSP levels in patients with T2DM. Although the change in GSP at week 12 was only close to a significant difference, the result suggested a potential possibility of significant changes in glucose metabolism in the case of a longer intervention period or larger doses.

However, as the primary outcome of our study, HbA1c showed no significant difference or change. The lack of change in HbA1c may be related to the relatively low HbA1c baseline levels and the intervention method (period, dosage, and administration method). First, in the meta-analysis, the reduction in HbA1c was found to be positively correlated with its baseline level (28). However, most of the participants recruited in this study had relatively low baseline HbA1c levels compared with those trials in the meta-analysis, and this factor may limit the effect of “maccog” TCM tea. Second, HbA1c reflects glucose metabolism in the past 2–3 months, but our intervention lasted for only 12 weeks (29). Considering that “maccog” TCM tea may take quite long to produce a marked effect, a prolonged intervention period may yield significant changes in HbA1c. Third, for better safety and acceptability, the substances applied in this study had a relatively small dosage and were consumed by drinking tea, which may affect the changes in HbA1c. Different effects on FPG and HbA1c were found in patients with T2DM who accepted different dosages of traditional Chinese herbal formula intervention with GQD (high dosage at 240 g and medium dosage at 144 g demonstrated significant reductions in changes in FPG and HbA1c levels, whereas placebo at 2.16 g and low dosage at 48 g did not) (10). Asai et al. conducted a mulberry leaf extract (DNJ) intervention of 12 weeks *via* tea drinking mode in patients with T2DM and found that 6 mg of DNJ did not significantly reduce the FPG and HbA1c levels compared with placebo (30); Hu et al. used a capsule (4.3 g) containing MFH such as corn stigma, mulberry leaves, and hawthorn intervention in patients with T2DM and found no statistical difference in FPG and HbA1c levels between IG and the placebo after 12 weeks of intervention (31). In our study, the total amount of “maccog” TCM tea was only 13 g. Whether a large dosage can produce more significant change in glucose metabolism indices in patients with T2DM should be explored in future work.

Apart from the effect on glycemic control, several substances of “maccog” TCM tea used in this study, including mulberry leaves, radix ophiopogonis, and gynostemma, have been shown to reduce blood lipid by reducing endogenous cholesterol synthesis, inhibiting the production of lipid peroxides, and clearing free radicals in the body (32–34). Dyslipidemia is one of the common problems in patients with T2DM, and the improvement in lipid profile will lead to remarkable benefits (35). The significantly lowered TC level after the intervention found in our study demonstrated a certain effect on blood lipid metabolism in patients with T2DM by “maccog” TCM tea. The improvement in blood lipid profile was also found in the research of Peng and Aramwit et al. (36, 37). Aramwit found that 12 weeks of mulberry leaf powder intervention can significantly reduce TG and LDL levels in participants with mild dyslipidemia (37). The changes in various parameters may be related to different participants. In our study, the decrease in TG in IG was slightly greater than that in CG, which was close to a statistical difference at week 12. A significant difference is likely with prolonged intervention period or increased dosage. Xu et al. showed that MFH intervention for 12 weeks can significantly increase HDL (10), but other studies found a decrease in HDL after MFH intervention for 12 weeks compared with placebo (18, 38). In our study, although the changes in HDL in IG were not statistically different from those in CG at week 12, we noted a decrease trend in IG and an upward trend in CG. Thus, the specific effects and reasons for the differences in human HDL changes of “maccog” TCM tea need to be further investigated.

Among the parameters of hepatorenal function, significantly lower TP, ALB, and CREA were found in IG than in CG. TP and ALB are closely related to protein metabolism, and their decrease generally indicates a malnourished status or reduced protein synthesis in the liver. The decrease in CREA is also a manifestation of body protein reduction. Whether the unexpected lower TP, ALB, and CREA after the intervention is related to the bitter taste of “maccog” TCM tea, which can affect the participants’ appetite, or due to some of the ingredients that affect protein metabolism is unknown and awaits further research. However, all hepatorenal indicators in our study were within the normal range, suggesting that taking these substances in tea drinking mode in short term did not cause abnormal human hepatorenal function. In addition, all participants did not have any significant adverse reactions during the study. Only a few participants had xerostomia or skin itching, and many reported beneficial changes in bowel movements, sleep, and vision. These results combined with good compliance confirmed the safety and feasibility of the intervention.

The gut microbiota and its metabolic products interact with the host in many different ways, influencing gut homoeostasis and health outcomes. The species composition of the gut microbiota has been shown to respond to dietary changes and then alter metabolic status (39). The results showed that after 12 weeks of “maccog” TCM tea consumption, the gut microbiota diversity index of patients with T2DM did not increase significantly, but the Ace and Chao1 indices in IG were slightly higher after the intervention, and the values were close to significant differences. We can speculate that the flora diversity index of patients with T2DM is very likely to increase significantly after the intervention with enlarged sample size.

The human gut microbiota contains more than a thousand species of which about 95% belongs to *Firmicutes* and *Bacteroidetes*, followed by *Actinobacteria* and *Proteobacteria* (40). Both IG and CG have similar relative abundances at the phylum level before and after the intervention, including *Bacteroidetes*, *Firmicutes*, *Proteobacteria*, and *Actinobacteria*. The results showed that *Proteobacteria*, *Firmicutes*, and *Bacteroidetes* in IG showed no significant difference compared with those in CG and the baseline, but *Actinobacteria* was significantly higher. In a study of a 23-week dietary intervention in 123 patients with central obesity, the participants’ abundance of *Actinobacteria* was significantly higher and that of *Proteobacteria* was significantly lower at 9 weeks and 23 weeks of intervention, respectively. The intervention was accompanied with improvements in inflammatory and metabolic indices (41). Although the participants and intervention project differed from our study, we noted similarities. Central obesity and T2DM are metabolic syndromes; the intervention of both studies included Chinese herbal medicines, and changes in the intestinal flora at the phylum level were basically consistent with our study, which suggested that *Actinobacteria* may be involved in the improvement of the metabolic syndrome. The abundance of *Actinobacteria*, *Lachnospiraceae*, *Bifidobacteriaceae*, *Phascolarctobacterium*, and *Bifidobacterium* after the intervention were significantly higher in IG than those at baseline. The abundance of *Lachnospiraceae* and *Phascolarctobacterium* after the intervention was significantly higher in IG than in CG. *Lachnospiraceae* can produce butyrate through the butyryl-CoA, acetate CoA-transferase, or butyrate kinase pathway (42). Butyrate can improve insulin production and sensitivity by stimulating glucagon-like peptide 1 (GLP-1) secretion and reducing inflammation of fat cells. Rising butyrate inhibits the development of low levels of chronic inflammation (43). Peng et al. found that *Lachnospiraceae* and *Clostridiaceae* increase significantly after 3 weeks by transferring gut microbes to non-obese diabetic mice, and this change was found to improve the insulin sensitivity of diabetic mice to some extent (44). *Bifidobacteriaceae* are a member of Gram-positive bacteria with intestinal barrier protection. They have been found to improve gut barrier function or reduce gut endotoxin level by stimulating the discharge of secretory immunoglobulin A (45). Le et al. found that oral administration of *Bifidobacterium* can reduce the expression levels of inflammatory adipocytokines and improve insulin resistance and glucose tolerance in obese mice (46). Cani et al. found that *Bifidobacterium* can improve the state of metabolism disorders in diabetic mice by reducing endotoxemia and intestinal permeability induced by a high-fat diet (47). Naderpoor et al. found that *Phascolarctobacterium* is positively related to insulin production and sensitivity through a survey of gut microbiome and insulin sensitivity in 38 obese patients (48). Our study concluded that the abundance of *Clostridiales* and *Lactobacillales* was negatively correlated with FPG levels, and the abundance of *Clostridiales* and *Lachnospiraceae* was negatively correlated with GSP levels; these results indicated that the higher the amount of beneficial bacteria, the more obvious the improvement in the glucose metabolism index. Furthermore, Pugliese et al. reported that obesity is associated with a change in gut microbiota composition characterized by a reduction in the abundance of *Bacteroidetes* and a proportional increase in *Firmicutes* (49). Obesity and T2DM are metabolic syndromes, which suggest that *Bacteroides*/*Firmicutes* may be involved in T2DM. In our study, the abundance of *Bacteroides*/*Firmicutes* was positively correlated with FPG and GSP levels.

In summary, “maccog” TCM tea can significantly improve the abundance of gut microbiota in patients with T2DM. These changes may promote the beneficial transformation of the intestinal environment through different modes of action, thereby improving the glucose metabolism of patients with T2DM.

This study had some limitations. First, the intervention used in this study was a combination of six substances, so we could not infer whether a single substance has a significant effect on improving glucolipid metabolism in patients with T2DM. Second, considering that the MFH materials used in this study are relatively common edible substances in daily life without special contraindications, syndrome differentiation and classification were not performed on the participants for the convenience of application. However, the improvements in patients with T2DM of different constitution types may vary. Third, due to practical constraint, this study was only carried out for 12 weeks and had no follow-up visits. Therefore, the long-term effect and safety of this formula on patients with T2DM need to be investigated in future studies for clarity. In general, fewer effective ingredients are extracted from tea than from decoction. Therefore, “maccog” TCM tea may not be as effective as decoction; with decoction, more positive therapeutic effects may be achieved with shorter intervention time under equal conditions.

## 5 Conclusions

The results of this study showed that “maccog” TCM tea for 12 weeks, which is safe and feasible, had some minor effects on improving glucolipid metabolism and significantly improved the abundance of gut microbiota in community patients with T2DM. The increase of beneficial bacteria abundance may be involved in the improvement in glucose metabolism indicators. More research is needed to confirm the positive effects and clarify its long-term effects and potential mechanisms.

## Data availability statement

The raw data supporting the conclusions of this article will be made available by the authors, without undue reservation.

## Ethics statement

The studies involving human participants were reviewed and approved by Ethics Committee of Soochow University. The patients/participants provided their written informed consent to participate in this study.

## Author contributions

LW, HM, and YZ designed the study. BH, JZ, ML, HY, JW, RG and JH conducted the study. BH and TY analyzed the data and wrote the first draft of manuscript. LW, HM and YZ supervised the study and reviewed and edited the manuscript. All authors have read and approved the final version of the manuscript.
