# Supplementary material for: Effect of “maccog” TCM tea on improving glucolipid metabolism and gut microbiota in patients with type 2 diabetes in community
Source: Front Endocrinol (Lausanne). 2023 Mar 8;14:1134877. doi: 10.3389/fendo.2023.1134877 (PMC10031008; doi:10.3389/fendo.2023.1134877)
Supplement: Supplementary file 1 [file DataSheet_1.pdf]

Supplementary Material

Part 1: Procedure for the formation of the "maccog" TCM tea formula

Firstly, on the basis of literature and books, we screened out the types of the traditional Chinese medicines (TCM) and their dosages. The inclusion criteria for the medicines to be included were as follows (1) being proved to have the hypoglycemic effect; (2) being inexpensive and easily accessible; (3) being commonly used and easily acceptable; (4) having no incompatibility with other medicines in the prescription; (5) being safe (harmless to the body with relatively low dose over a long period). According to the above principles, 15 kinds of TCM were firstly selected out, which included mulberry leaf, fructus lycii, gynostemma, radix astragali, radix ophiopogonis, cortex eucommiae, hawthorn, cortex lycii, corn stigma, radish seed, Chinese yam, Indian buead, lobed kudzuvine root, coix seed and moringa seed, with a proposed dose of 10–15 g for each. The first 10 types of medicines were prescribed to be directly soaked in boiling water, and the last 5 were grounded into powder before soaking in boiling water.

Secondly, according to consultation results with five experts in TCM, we made some adjustments according to suggestions. The first change was to remove five medicines (Chinese yam, Indian buead, lobed kudzuvine root, coix seed and moringa seed) to enable a unified application style medicines were applied in a same way by soaking in boiling water for easier handling and better compliance. Although the tea drinking style may limit the extraction of some active ingredients of TCM, it is safer with less side effects and without special taboos based on experience. The second change was to remove radish seed and cortex eucommiae with the reason that diarrhea was reported in some clinical patients after taking radish seed and increased blood glucose level was found after using cortex eucommiae in a few animal experiments although ~~reducing~~ blood sugar in most studies. The third change was the daily doses of the medicines should be reduced appropriately for easy handling in tea drinking mode. According to the opinions of experts, the type and dosage of medicines are determined to be: 2 g mulberry leaf, 2 g gynostemma, 2 g corn stigma, 2 g radix astragali, 2 g cortex lycii, 3 g radix ophiopogonis, 3 g fructus lycii and 8 g hawthorn. In addition, according to the recommendation of the experts, all TCM are scheduled to be purchased from Suzhou Tianling Traditional Chinese Medicine Slices Co., Ltd. (Brand Li Liangji, a century-old famous brand of TCM with a good reputation in Suzhou) to ensure the safety and reliability of the quality of the medicine.

Thirdly, 8 patients with T2DM were recruited to participate the pre-trail application. All patients were instructed to taking the prescribed medicines in tea drinking style for one week. No one reported adverse reactions, but three reported worries about the increases in blood glucose by hawthorn and fructus lycii because of their sweet taste. These two medicines were removed and the final formula of MFH thereby included 2 g mulberry leaf, 2 g corn stigma, 2 g radix astragali, 2 g gynostemma, 2 g cortex lycii and 3 g radix ophiopogonis.

By taking the first letter of each medicine, we named the formula as "maccog" TCM tea (**m**ulberry leaf, radix **a**stragali, **c**orn stigma, **c**ortex lycii, radix **o**phiopogonis, and **g**ynostemma).

Part 2: Figure S1.

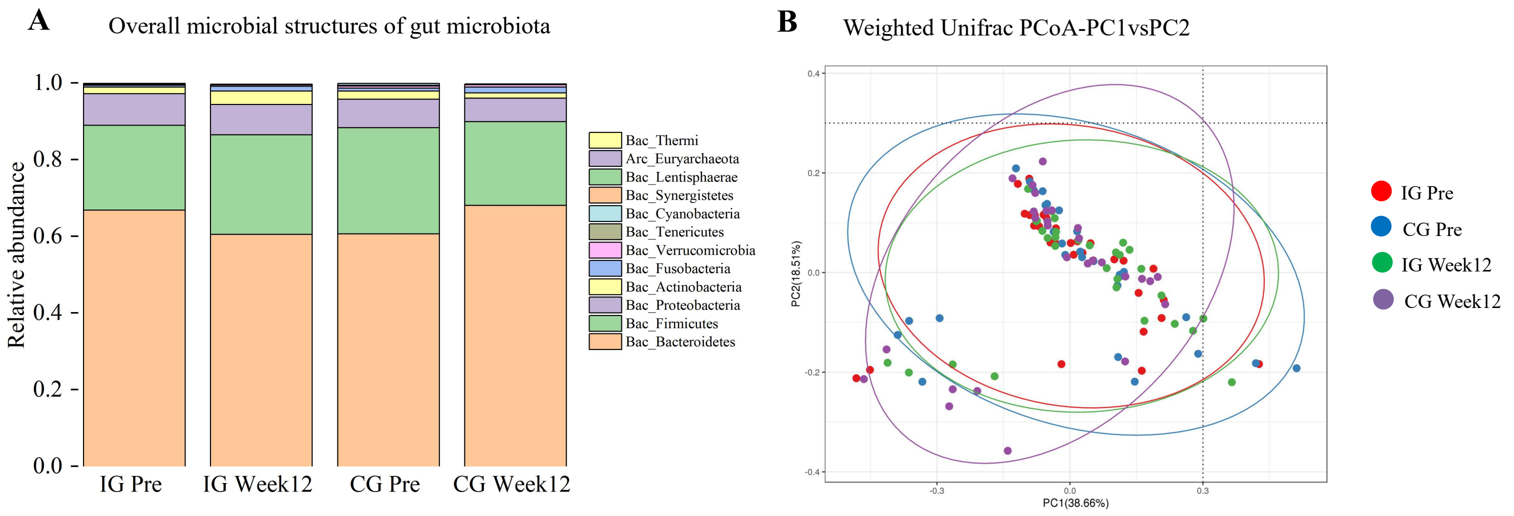

Figure S1. Overall microbial structures and beta diversity of gut microbiota in IG and CG. (A) Overall microbial structures of gut microbiota. (B) Weighted Unifrac PCoA-PC1vs. PC2. CG, control group; IG, intervention group.
